# Human social isolation and stress: a systematic review of different contexts and recommendations for future studies

**DOI:** 10.47626/2237-6089-2021-0452

**Published:** 2024-02-28

**Authors:** André Comiran Tonon, Ana Carolina O. V. de Abreu, Mariana Mendonça da Silva, Patrice de Souza Tavares, Fernanda Nishino, Paula Versignassi, Guilherme Rodriguez Amando, Débora Barroggi Constantino, Luísa Klaus Pilz, Eduardo Steibel, Deborah Suchecki, Fernanda Gaspar do Amaral, Maria Paz Hidalgo

**Affiliations:** 1 Laboratório de Cronobiologia e Sono HCPA UFRGS Porto Alegre RS Brazil Laboratório de Cronobiologia e Sono, Hospital de Clínicas de Porto Alegre (HCPA), Universidade Federal do Rio Grande do Sul (UFRGS), Porto Alegre, RS, Brazil.; 2 Programa de Pós-Graduação em Psiquiatria e Ciências do Comportamento UFRGS Porto Alegre RS Brazil Programa de Pós-Graduação em Psiquiatria e Ciências do Comportamento, UFRGS, Porto Alegre, RS, Brazil.; 3 Programa de Pós-Graduação em Psicologia LPNeC UFRGS Porto Alegre RS Brazil Programa de Pós-Graduação em Psicologia, Laboratório de Psicologia Experimental, Neurociências e Comportamento (LPNeC), UFRGS, Porto Alegre, RS, Brazil.; 4 Laboratório de Neurobiologia da Pineal Departamento de Fisiologia UNIFESP São Paulo SP Brazil Laboratório de Neurobiologia da Pineal, Departamento de Fisiologia, Universidade Federal de São Paulo (UNIFESP), São Paulo, SP, Brazil.; 5 Departamento de Psicobiologia UNIFESP São Paulo SP Brazil Departamento de Psicobiologia, UNIFESP, São Paulo, SP, Brazil.

**Keywords:** Psychiatry, depression, anxiety, lockdown, social distancing, social connection

## Abstract

**Objectives:**

The emergence of the coronavirus disease 2019 (COVID-19) pandemic and subsequent lockdowns and social distancing measures adopted worldwide raised questions about the possible health effects of human social isolation.

**Methods:**

We conducted a systematic review on PubMed, Scopus, and Embase electronic databases using terms related to human social isolation – defined as the isolation of an individual from regular routines and usual social contact – and psychological stress, searching for simulated or naturalistic isolation environments. We present the main results, as well as the validity and limitations of each model. PROSPERO registry number: CRD42021241880.

**Results:**

Despite the diversity of contexts reviewed, some outcomes almost ubiquitously relate to psychological stress, i.e., longer periods, expectation of a longer period, confinement, lack of social interaction, and support. Based on the results, and considering that most studies were not designed for the purpose of understanding isolation itself, we propose a group of recommendations for future experimental or naturalistic research on the topic.

**Conclusion:**

Evidence on the impact of different situations in which individuals are subjected to social isolation can assist in development of directed preventive strategies to support people under similar circumstances. Such strategies might increase the general public’s compliance with social distancing as a non-pharmacological intervention for emerging infectious diseases.

## Introduction

Social connection (i.e., the structural, functional and qualitative aspects of social relationships) is a major determinant of psychological and emotional well-being among humans.^1,2^ A consistent body of evidence supports the notion that satisfying social relationships and interactions are vital for neuropsychomotor development^3,4^ and maintenance of mental and physical health.^5,6^ In addition, the dynamics of social contacts contribute to establishing a social schedule that may influence daily patterns of light exposure, which is the main environmental cue that synchronizes biological rhythms to the environment.^7,8^ Social stimuli also play a key role in non-photic entrainment of biological rhythms by determining the timing of other non-photic entrainers, such as exercise and mealtimes.^9,10^

Some situations require that individuals be isolated from their normal routine and social contact, such as work missions in extreme environments, as is the case of the Antarctic continent, or participating in a space exploration trip. Moreover, strategies for containment of air-born infectious diseases also require that individuals be forced into isolation or social distancing, like severe acute respiratory syndrome-Middle East respiratory syndrome (SARS-MERS) and, more recently, the COVID-19 pandemic.^11^ In spite of the major characteristics that shape each context, isolating oneself is considered a stressor and, therefore, has predictable consequences. Similarities among distinct isolation contexts have already been described, including modeling of spaceflight missions in Antarctic environments^12^ and basing the theoretical approach to prolonged isolation and confinement of COVID-19 lockdowns on spaceflight analogs.^13^

As for the literature on human beings, social isolation may be objective (e.g., living alone) and/or perceived and subjective (e.g., feeling lonely). Both types of social isolation have been associated with poorer sleep quality, impaired cognitive function, and mental health problems,^14^ as well as with unhealthy behaviors such as smoking and physical inactivity.^15^ Socially isolated individuals also exhibit adverse outcomes related to physical well-being, mostly due to isolation-induced stress response. These include increased blood pressure, impaired C-reactive protein and lipid profiles, poorer immune response, higher risk of developing Alzheimer’s disease, and increased overall mortality.^14-16^

Evidence regarding the impact of social isolation on human beings is not abundant. Hence, concepts are not well defined and causality is difficult to establish. This creates a heterogeneous body of evidence and makes it hard to understand the independent effect of social connectedness and isolation. Notwithstanding, several documented outcomes indicate that social isolation exerts its effects by modulating the stress response. Therefore, the aim of this study was to systematically review the available evidence on the effects of human social isolation, defined here as the isolation of an individual from regular routines and usual physical social contact, on psychological stress, including emotional, behavioral and cognitive impairment, and sleep problems. Even though perceived loneliness, social disconnectedness/alienation and marital quality are also described as dimensions of social connections, these concepts were not included in the present review. In addition, based on the results of this review, we aim to compile a list of essential elements to be incorporated in future research on the topic.

Evidence on the impact of different situations in which individuals are subjected to social isolation can assist in development of directed preventative strategies to support people under similar circumstances. Such information might also help healthcare professionals track possible health outcomes in socially isolated people.^17,18^

## Methods

### Search method and eligibility criteria

The present systematic review was registered in the PROSPERO database (number CRD42021241880). We conducted a systematic review of the literature on PubMed, Scopus, and Embase electronic databases using terms related to social isolation and psychological stress (full search terms available in Supplementary Material S1, available online-only).

We searched for peer-reviewed papers reporting empirical studies including data on social isolation as a factor. We defined “social isolation” as isolation of an individual from regular routines and usual physical social contact, associated with confinement or not. The settings of interest were simulated isolation environments or naturalistic environments, including home seclusion, quarantine, and space flight. Studies on isolation as synonyms of “loneliness” or social reclusion (e.g., *hikikomori* or elderly people in geriatric homes) were not eligible for this review. We also excluded studies of individuals with unspecified isolation onset, of physical distancing only (e.g., distancing for the purpose of prevention of transmission of infectious diseases), and studies that aimed to study other major stressors (e.g., natural disaster, public health threat) without accounting for isolation.

Given the heterogeneity of the literature on this topic, we included all studies with at least one outcome related to psychological stress in a situation of isolation. These included subjective psychological stress, tension, irritability, anxiety, depression, and sleep problems. In addition, due to the restricted number of empirical studies, we did not limit the number of subjects included in the studies, thereby avoiding omitting information from small sample studies. Some contexts of interest inevitably recruit a small number of subjects, e.g., space flights, experimental isolation, and Antarctic winter-over cohorts. We only included studies with non-clinical human populations described in articles written in English, Portuguese, Spanish, French, or German. All animal models were excluded. Thus, we retained only observational studies (i.e., cohort and cross-sectional study designs) and quasi-experimental studies. Randomized clinical trials and review studies were also excluded. Looking for the highest methodological quality, we excluded non peer-reviewed references and unpublished data.

**Figure 1:**
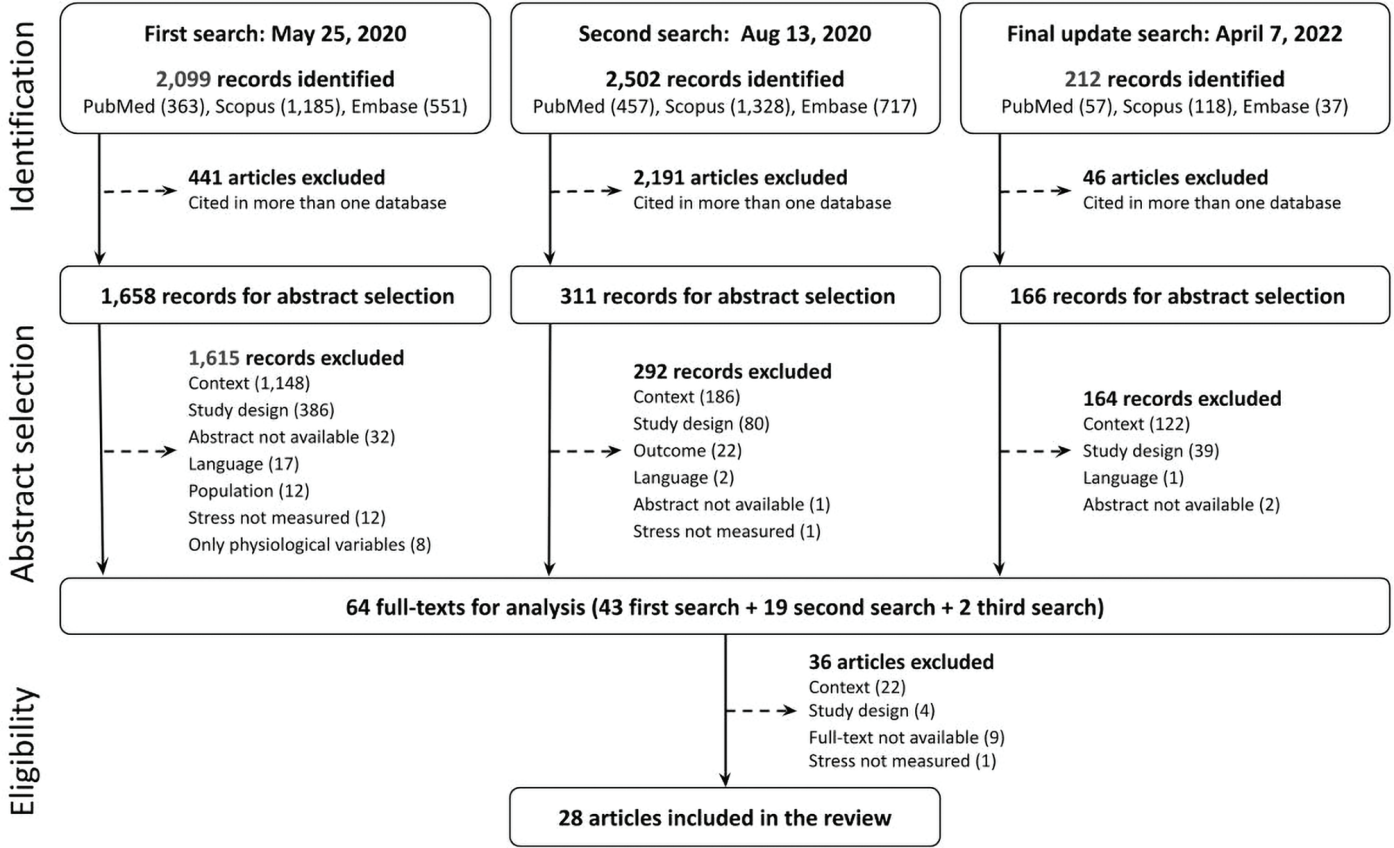
Diagram of the systematic review process

The first search was completed on May 25, 2020, retrieving 2,099 records and resulting in 1,658 abstracts after exclusion of duplicates. To identify newly published articles on isolation in the context of severe acute respiratory syndrome coronavirus 2 (SARS-CoV-2) pandemic, we conducted a second search on Aug 13, 2020, retrieving 311 new records. Compared to 2019, the number of abstracts identified in 2020 increased 22.4-fold (from 32 to 716) on PubMed, 17.8-fold (from 43 to 765) on Embase, and 19.7-fold (from 23 to 454) on Scopus. Compared to the same year, the number of abstracts identified in 2021 increased 44-fold on PubMed (1,405 in 2021) and Embase (1,895 in 2021), and 20-fold (460 in 2021) on Scopus (Figure S1, available as online-only supplementary material). Considering the notable increase in abstracts because of the COVID-19 pandemic, a third search was conducted on April 7, 2022 to update the search with studies conducted in contexts not related to COVID-19 (thus excluding keywords related to COVID-19) retrieving another 166 studies (see Discussion, for more information). Figure 1 illustrates the systematic review process.

Two authors (ACT and FAN) independently and blindly performed the search and screened abstracts against eligibility criteria. Any disagreement between these authors was resolved by consensus with a third author (PST). Another six researchers were divided into pairs and blindly read in full all papers initially selected.

### Data collection and quality assessment

This study was conducted according to recommendations made by Mueller et al.^19^ Two independent researchers retrieved a standardized set of information from all full texts included in the review. A third independent researcher matched the two independent reviews and solved any disagreements via consensus. The data collection phase included assessment of methodological quality, performed using the modified Newcastle-Ottawa Scale (NOS)^20^ for cross-sectional or cohort studies. This instrument lists key methodological aspects of observational studies and scores high-quality studies with a “star” for each aspect. A maximum of eight or nine stars can be given to cohort or cross-sectional studies, respectively. The quality of studies is analyzed in terms of sample selection (maximum of three stars for cohorts or four stars for cross-sectional studies), comparability (maximum of two stars) and outcome assessment (maximum of three stars).

## Results

**Figure 2:**
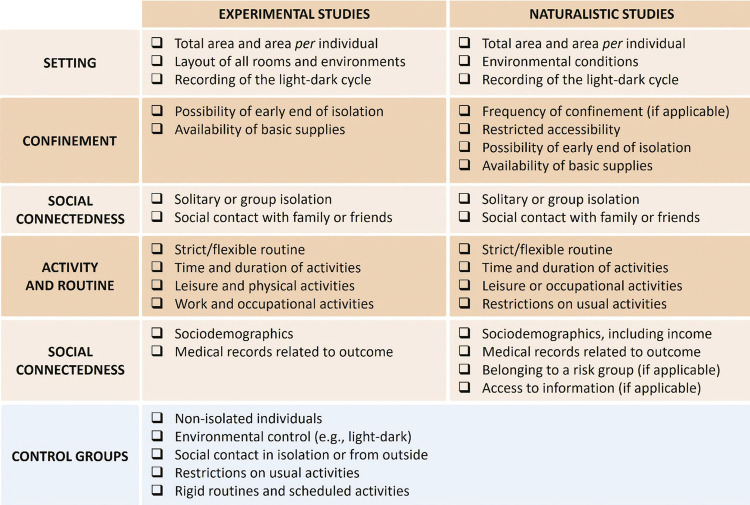
Recommendations for future research on human isolation

**Figure 3:**
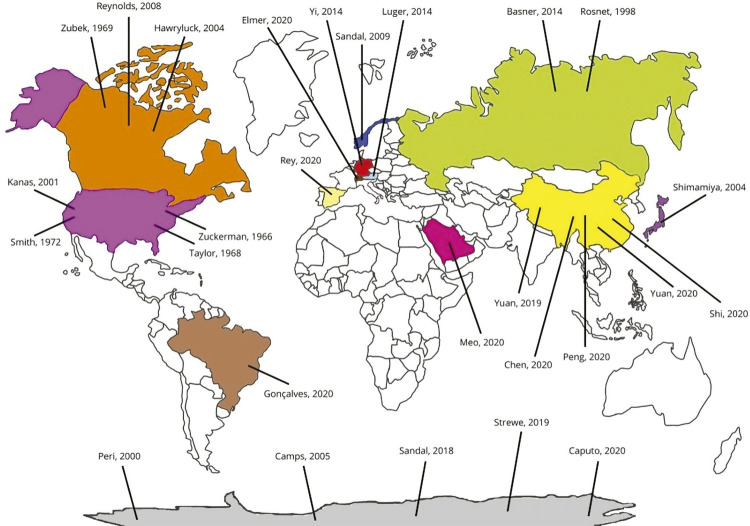
World map illustrating the setting locations of the included studies

Data from the studies included and the results of the assessment of quality of evidence are shown in Table 1. The studies’ contexts are described in terms of duration and other relevant characteristics related to environmental control, confinement, social connectedness, activities, and routine (see Figure 2 for details). The following section summarizes the findings of the 28 studies included in the review grouped according to the model of isolation (see Figure 3 for a visual representation of the setting location of the included studies). A summary of the main outcomes of each study is shown in Table S1 and Supplementary Material S2, available as online-only supplementary material.

**Table 1:** General characteristics of included studies and quality assessment

	Context			Sample		Study design	Quality		
Reference	Model	Duration	Other characteristics	n	Charac- teristics		Selection	Comparability	Outcome
Hawryluck et al., 2004^21^	Pandemics	Median of 10 (8-10) days	66% in home quarantine, 34% in work quarantine; 58% remained inside their residence for the duration of quarantine; description of personal protective measures; limited social contact; social distancing and restrictions on daily activities; no description of routine and basic supplies.	129	Quarantined people; most with a high level of education; 68% were healthcare workers.	Cross-sectional	★★	★★	★★
Reynolds et al., 2008^22^	Pandemics	Mean of 8.3 ± 3.1 days	89.8% in home quarantine, 10.2% in work quarantine; no description of isolation frequency; 15.8% compliant with all protective measures; no description of social distancing; limited social contact; restrictions on daily activities; no description of routine and basic supplies.	1,057	Quarantined people; Mean age 49.2 years (SD = 15.7).	Cohort	★	★★	★★
Yuan et al., 2020^23^	Pandemics	14 days	Home quarantine; no description of isolation frequency, personal protective measures, social distancing, social contact, restrictions on daily activities, routine, or basic supplies.	939	Quarantined people; 65.92% were university students.	Cross-sectional	★★★	★	★★
Meo et al., 2020^24^	Pandemics	14 days	Home quarantine; no description of isolation frequency, personal protective measures, social distancing, social contact, restrictions on daily activities, routine, or basic supplies.	530	Medical students.	Cross-sectional	★★★	★	★★
Gonçalves et al., 2020^25^	Pandemics	Varies among participants	Home quarantine; no description of isolation frequency, personal protective measures, social distancing, social contact, restrictions on daily activities, routine, or basic supplies.	539	Quarantined people; aged from 18 to 76 years (M = 37.04; SD = 12.91).	Cross-sectional	★★★	★	★★
Shi et al., 2020^26^	Pandemics	Unspecified	Home quarantine; no description of isolation frequency, personal protective measures, social distancing, social contact, restrictions on daily activities, routine, or basic supplies.	56,679	Quarantined people; most with high level of education.	Cross-sectional	★★★★	★★	★★
Rey et al., 2020^27^	Pandemics	Unspecified	Home quarantine. 27.9% of the participants had been confined at home for more than 5 days, 14.8% for 4 days, 12.2% for 3 days, 9.3% for 2 days, 12.4% for 1 day, and 21.6% for no days. No description of personal protective measures. Description of social distancing. Limited social contact. Restrictions on daily activities. No description of routine. Fear of food or health products shortages.	3,055	Quarantined people; most well educated; 75.1% were women.	Cross-sectional	★★★★	★★	★★
Chen et al., 2020^28^	Pandemics	At least 2 months	Home quarantine; no description of isolation frequency, personal protective measures, social distancing, social contact, restrictions on daily activities, routine, or basic supplies.	992	Chinese college students; Mean age = 19.45 ± 1.41 years.	Cross-sectional	★★★	★	★★
Peng et al., 2020^29^	Pandemics	14 days	Home quarantine; no description of isolation frequency, personal protective measures, social distancing, social contact, restrictions on daily activities, routine or basic supplies.	2,237	Quarantined people; aged from 18 to 70 years without clear diagnoses of COVID-19 infection.	Cross-sectional	★★★★	★★	★★★
Elmer et al., 2020^30^	Pandemics	14 days after introduction of social distancing	Home quarantine; no description about frequency and personal protective measures; social distancing and restrictions on daily activities; limited social contact; no description of routine and basic supplies.	336	Quarantined people; college students; higher proportion of male students.	Cohort	★	★	★★
Yi et al., 2014^31^	Space	520 days	Simulated trip; no environmental challenges; light-dark cycle not reported; limited occupational activities; strict routine; contact with family or friends not reported; usual basic supplies; possibility of early end of isolation not reported.	6	Males, mean age of 33 years.	Cohort	★★	★★	★★★
Luger et al. 2014^32^	Space	Mean of 23.9 ±9.0 (isolated) or 18.4 ± 6.7 days (control)	Simulated trip; environmental challenges not reported; light-dark cycle not reported; no record of leisure/occupational activities; no record of routine; contact with family or friends not reported; no record of basic supplies; possibility of early end of isolation not reported.	28	Isolated group: four females and 10 males, mean age of 32.9 ± 9.2 years. Control group: four females and 10 males, mean age of 32.4 ± 12.3 years.	Cohort	★★★	★	★★
Yuan et al., 2019^33^	Space	180 days	Simulated trip; environmental challenges not reported; light-dark cycle of 24 hours and 40 minutes from day 72 to day 108; presence of leisure/occupational activities; strict routine; limited social contact with family and friends; usual basic supplies; possibility of early end of isolation not reported.	4	3 males and 1 female, mean age of 34.2 ± 6.6 years	Cohort	★★	★★	★★
Basner et al., 2014^34^	Space	520 days	Simulated trip; space-like environment with altered light-dark cycle; presence of leisure/occupational activities; strict routine; no report of social contact with family and friends; limited basic supplies; possibility of early end of isolation not reported.	6	Males, mean age of 32 years (range 27-38).	Cohort	★	★★	★★
Kanas et al., 2001^35^	Space	4-7 months	Official trip; space environment with altered light-dark cycle; leisure/occupational activities or routine not reported; no report of social contact with family and friends; no report of basic supplies; early end of isolation not possible.	71	Five U.S. astronauts, eight Russian cosmonauts and 42 U.S., and 16 Russian control personnel.	Cohort	★★	★	★★
Rosnet et al., 1998^36^	Space	135 days	Simulated trip; environmental challenges not reported; light-dark cycle not reported; limited leisure/occupational activities; strict routine; contact with family or friends not reported; no record of basic supplies; possibility of early end of isolation not reported.	3	Males aged 31-36 year.	Cohort	★★	★	★★
Sandal et al., 2009^37^	Submarine	10 or 40 days	NATO standard submarines, environment with altered light-dark cycle; leisure/occupational activities or routine not reported; no report of social contact with family and friends; no report of basic supplies; early end of isolation not possible.	196	39 submarine workers on a 40-day (mean age of 27.11 ± 4.97 years) or 10-day (28.30 ± 3.78) trip; 25 office workers (32.23 ± 5.61 years); 121 military recruits (20.3 0± 4.30 years).	Cohort	★★★		★★
Décamps et al., 2005^38^	Antarctica	350 days (50 weeks)	Restricted accessibility. Early end of isolation not possible during winter. Group isolation. Limited social contact with friends or family. Exposure to extreme environmental conditions. Altered light-dark cycle. No description of availability of basic supplies. No description of routine. Presence of occupational activities. Limited leisure activities.	27	Average age = 29 years and 7 months (range = 21 to 59 years).	Cohort	★		★★★
Per et al., 2000^39^	Antarctica	2-5 months	Restricted accessibility. Possibility of early end of isolation. Group isolation. No description of social contact with family or friends. Exposure to extreme environmental conditions. Altered light-dark cycle. No description of availability of basic supplies. No description of routine. Presence of occupational activities. No description of leisure activities.	11	Male volunteers. Ages 37-51 years.	Cohort	★		★★
Sandal et al., 2018^40^	Antarctica	10 months	Restricted accessibility. Early end of isolation not possible. Group isolation. Limited social contact with family or friends. Exposure to extreme environmental conditions. Altered light-dark cycle. No description of availability of basic supplies. No description of routine. Presence of occupational activities. Limited leisure activities.	27	Crew 1: males; ages ranging from 23 to 58 years (M = 38.3, SD = 10.64). Crew 2: 10 males and three females; ages ranging from 22 to 51 years (M = 34.5, SD = 9.17).	Cohort	★		★★
Strewe et al., 2019^41^	Antarctica	12 months	Restricted accessibility. Early end of isolation not possible during winter. Group isolation. Limited social contact with family or friends. Exposure to extreme environmental conditions. Altered light-dark cycle. Limited basic supplies. No description of routine. Presence of occupational activities. No description of leisure activities.	26	10 females and 16 males. Females aged (31.8 ± 6.1) and males aged (37.7 ± 9.1). Expeditioners were primarily employed as scientists, cooks, engineers (including IT), electricians, and medical doctors.	Cohort	★	★	★★
Caputo et al., 2020^42^	Antarctica	12 months	Restricted accessibility. Early end of isolation not possible during winter. Group isolation. Limited social contact with family or friends. Exposure to extreme environmental conditions. Altered light-dark cycle. Limited basic supplies. No description of routine. Presence of occupational activities. No description of leisure activities.	13	Healthy volunteers (10 men, three women, average age 34.1 ± 3.1, range 24-56 years).	Cohort	★	★	★★
Tortello et al., 2020^43^	Antarctica	12 months	Restricted accessibility. Early end of isolation not possible during winter. Group isolation. Limited social contact with family or friends. Exposure to extreme environmental conditions. Altered light-dark cycle. Limited basic supplies. No description of routine. Presence of occupational activities. No description of leisure activities.	13	Healthy volunteers (men age 34 ± 1, similar anthropometric characteristics (body mass index: 26 ± 1 kg/m2).	Cohort	★	★	★★
Shimamiya et al., 2004^44^	Experimental/ lab conditions	10 days	Physical space of 34.1 m^2^ (6.82 m^2^ per participant). Group isolation. No description of social contact with family or friends. Possibility of early end of isolation . No description of availability of basic supplies. No description of light-dark cycle. No description of routine. Limited occupational activities. No description of leisure activities.	10	Male university students (age 20-27 years, mean 22.8).	Cross-sectional	★★		★★
Smith et al., 1972^45^	Experimental/ lab conditions	21 days	Physical space of either ~2 m^3^ or ~5.7 m^3^ of usable space per participant. Group isolation. No contact with family or friends. Possibility of early end of isolation. Usual basic supplies. No description of light-dark cycle. Flexible routine. Limited occupational activities. Limited leisure activities.	56	Volunteer Naval enlisted men; ages ranging from 18 to 32, averaging 20.8 years.	Quasi-experimental	★	★★	★★
Zubek et al., 1969^46^	Experimental/ lab conditions	7 days	Physical space of 2.1 m in height, 2.7 m in diameter, and 2.3 m at the base (dome-shaped). Solitary isolation. Limited or absent social contact with family or friends. Possibility of early end of isolation. No description of availability of basic supplies. Unaltered light-dark cycle. Flexible routine. Limited occupational activities. Limited leisure activities.	66	Male university students.	Quasi-experimental	★★	★★	★
Taylor et al., 1968^47^	Experimental/ lab conditions	8 days	Physical space of 13.4 m^2^ (6.7 m^2^ per participant). Group isolation. No social contact with family or friends. Possibility of early end of isolation. Limited basic supplies. Altered light-dark cycle. Flexible routine. Limited occupational activities. Limited leisure activities.	168	Males. Ages ranging from 18 to 20.	Cross-sectional	★★	★★	★
Zuckerman et al., 1966^48^	Experimental/ lab conditions	Two sessions of 8 hours and 5 minutes, one week apart	No description of size of physical space. Solitary isolation. No contact with family or friends. Possibility of early end of isolation. Altered and unaltered light-dark exposure. Strict routine. Absence of occupational activities. Absence of leisure activities.	18	Healthy males.	Quasi-experimental	★	★	★

NATO = North Atlantic Treaty Organization. SD = standard deviation.

A series of studies included in this review report that the longer people stay in isolation, the higher the incidence of adverse psychological outcomes (or the worsening of existing ones). Of note, one experimental study^47^ demonstrated that those individuals expecting longer stays (in this context, 20 days) were more prone to report anxiety and stress than those who expected shorter missions (4 days). Another experiment with perceptual restriction in women found an increase in anxiety with the lengthening of isolation for more than 6 hours.^48^ None of the Antarctic trip articles focused on studying duration, but it is notable that in the summer campaign,^39^ in which participants only remain isolated for 2-5 months, the outcomes (e.g., mood and coping strategies) were much more positive than in winter-over studies (10-12 months). However, several other factors might play a role in these associations, such as seasonal conditions, crew rotation, and outdoor activities. Finally, most studies of pandemics showed that duration of quarantine significantly increased stress and PTSD symptoms,^21,22,29^ but one study did not corroborate these results.^25^

A peculiar “third quarter effect” was described in some of the studies. This phenomenon is described as the surge of psychological and interpersonal difficulties (e.g., sleep difficulties, depressive symptoms, irritability, worsening in performance) after the halfway point of missions, regardless of the actual length of these missions.^49^ It has been described in several settings of isolated and/or confined subjects and in extreme environments, such as Antarctic and submarine expeditions, space missions and space analogs. This phenomenon was reported in some of the space trip studies,^32,33,50^ one submarine experiment,^51^ and two Antarctic expeditions.^38,40^ However, a study conducted by Décamps and Rosnet^38^ describes a significant decrease in the number of somatic reactions from the third quarter of the stay on, indicating a positive third quarter phenomenon.

Physical isolation may also be accompanied by a disconnection of social contact with family, friends, and significant others. Some of the studies included in the present review identified this aspect in their samples. Evidence from experimental isolation shows that individuals with a possibility of social contact presented less conflicts and irritation towards others.^45^ Another experimental study described temporal disorientation, feelings of hostility, and loneliness to be particular to socially isolated individuals, compared to a confined group with social contact and an ambulatory control group.^46^ One experiment reported that subjects with outside verbal stimulation were less stressed than subjects under perceptual isolation.^47^ In a cross-sectional study of isolation due to pandemics, satisfaction with the quality and number of online social interactions associated positively with well-being and negatively with stress.^25^ Another study, conducted with students during the COVID-19 crisis, reports that individuals isolated from their personal network reported an increase in depressive symptoms, while individuals interacting less with their personal network were more anxious.^30^ In the same study, students who reported greater emotional support were less depressed and felt less lonely. In one Antarctic study, researchers found a decline in social interaction as the expedition progressed, and also a positive correlation between social support and stress recovery.^43^

Finally, we noted only a few references to physical space and crowdedness. Overall, these are underreported features that have proved to be significant in a few studies. For instance, crowding was reported to be a factor that contributed to subjective stress and anxiety, indicating better adaptation to confinement among groups of few people.^45^ Moreover, people in more crowded spaces tend to be more annoyed with physical features of the laboratory setting. One study of quarantined individuals analyzed the size of the residence, indicating that those living in larger (i.e., more than 120 m^2^) and less crowded (i.e., one or two, as opposed to three or four people) residences showed lower psychological impact, stress, anxiety, and depression.^27^ In addition, people living in a residence with access to open-air space showed slightly better psychological outcomes.

Notably, the contexts of isolation included in this review were markedly distinct. The naturalistic studies presented here were situations of isolation – and often confinement – that were not designed for the purpose of understanding isolation itself. Nevertheless, some common reports have been described that might apply to more than one of these contexts, thus representing common repercussions of prolonged isolation apart from other stressors. Henceforth, it is of utmost importance that we define the strengths and limitations of each model reviewed here, so that we are able to delineate directions for future research on the topic.

## Discussion

### Strengths and limitations

#### Experimental and quasi-experimental models

Experimental and quasi-experimental studies designed to investigate isolation are typically conducted in highly controlled setups, which allow researchers to look into the relationship of isolation with specific stress factors such as social contact,^46^ duration expectancy,^47^ and crowdedness and compatibility.^45^ In addition, given that (a) the studies are conducted in research facilities, (b) the duration of the isolation is often known, (c) early end of isolation is an option, and (d) close supervision occurs, feelings of fear tend to be more seldom than in naturalistic models (which, again, allows investigation of interaction between specific stressors). The number of participants in experimental and quasi-experimental models tends to be small (from 10 to 168 subjects) and, in the case of the five articles included, most subjects were young and all were male. Besides, since volunteers are recruited specifically for the studies, they often have little to do during the isolation period (occupational and leisure activities are either limited or absent), with the exception of a few tests. Therefore, the main advantage of experimental models would be the possibility of investigating the effects of isolation on its own or combined with specific stressors. Disadvantages include lack of external validity and samples that are not representative of the general population.

It is important to note that studies classified as “experimental and quasi-experimental” can be vastly different from each other. However, because the chambers to which participants of experimental studies are allocated are typically small and the effects of crowdedness and lack of privacy can be investigated in studies of group isolation, it is possible to draw a parallel with the situation of people living in environments such as small apartments and/or with many family members or housemates.

#### Space missions and analogs

Studies regarding space flights resemble the experimental designs described above in a series of aspects. Of the six studies included in this review, only one studied a cohort from an official trip, while the remaining five are realistic experimental simulations of space environments. This means that apart from the setting design (i.e., a terrestrial analog site *versus* an experimental chamber that looks like a house), both environments are extremely controlled. The settings of space analogs, however, are generally poorly described. For example, only three studies report environmental challenges including altered light-dark cycle. Reporting of social connectedness and daily routines is also unsatisfactory or nonexistent. Even so, the major issue of such models for the purpose of understanding the impact of social isolation is the lack of external validity regarding sample selection. All studies recruited astronauts or cosmonauts, who acknowledge and accept confinement in aircrafts and space shuttles as part of their profession. These volunteers are mostly healthy, highly educated, men with previous training who underwent rigorous selection from among other candidates.

The usefulness of space analog missions as possible models of social isolation and distancing seem to resemble that of an experimental confinement. The addition of a space-like environmental control (e.g., pressure and light exposure) could be useful for the study of isolation in official space missions, but makes no contribution to understanding of the psychological impacts of isolation per se. Apart from that, naturalistic studies of official flights could benefit from a more detailed characterization of environment, routines, and social connectedness.

#### Submarines

The submarine missions resemble space analogs in terms of confinement and recruitment of crew members. We observe a cohort mostly composed of young male individuals with limited external validity. Once again, it seems like the usefulness of a submarine model of isolation is narrowed to initiatives of experimental studies of confinement in small groups of people. As described above, several methodological aspects (e.g., environmental control and social connectedness) could be applied to better characterize the context of isolation.

#### Antarctic

The Antarctic cohorts are similar to those of space flight studies, since they tend to be small and mostly composed of highly educated male volunteers. Participants are rigorously selected and trained in order to be prepared for difficult situations, hence results of such studies likely underestimate the reactions many people would present in moments of unexpected social isolation. Extreme environmental conditions prevent access to the Antarctic stations during winter, making an early end of isolation impossible, even if desired or needed, and therefore reassuring the isolation condition (which can be dubious in pandemic studies). The light-dark cycle, barometric pressure, and outside air temperature are additional stress factors. Communication with the outside world is limited and may temporarily break down due to extreme environmental conditions, but in general – and especially in recent years – it is possible to maintain social contact with family and friends by phone, email, video calls, and social media, which is especially important for maintenance of mental health.^52^ Due to the impossibility of an early end of isolation , possibility of virtual contact and presence of small groups in the same environment, we suggest that Antarctic studies can be used as models for the study of lockdown situations. However, differences include the environmental conditions and the fact that, in lockdowns, it is possible to leave the house in cases of extreme need.

#### Pandemics

An important limitation found in pandemic-related work is that most studies improperly report or do not report the length of social isolation. Few studies present a direct question about how many days the respondents had been in social isolation or whether they left the house for some reason. Most of them just assumed that study subjects complied with the government-implemented measures of social isolation and distancing, which may not be a reliable datum, making it difficult to be sure for how long and at what frequency individuals were isolated (e.g., all of the time, most of the time, sometimes or not in social isolation). The characteristics of isolation are, in general, poorly described and can vary greatly among the participants of a single study and between different studies within this model. Variable aspects include the kind of isolation (home or work, alone or with family members), the possibility of leaving the house for some activities (going to the market) and the number of face-to-face contacts. Another fact to take into account is that in some samples people were coerced, with strict measures, to stay in social isolation while in others they were only requested to stay in social isolation.

Generally, the samples are not totally representative of the population, with a higher percentage of women, adults, and young adults (ages between 18 to 45 years old), healthcare workers and students, and people with high levels of education. Most studies were designed in the form of online questionnaires and took place in big cities, with a tendency to be places with a larger number of disease cases. These are factors that by themselves can restrict the sample and have an effect on the results.

When dealing with a pandemic moment, several factors can influence the psychological wellbeing other than the isolation itself.^18,53^ The fear of getting infected or infecting others, emergence of financial issues, and dissatisfaction with the information received about the current situation are some of the other stressors affecting these populations.^27^ There is scant information regarding people’s routines, light exposure, access to basic needs, exercise, and leisure activities. With the studies’ results in mind, we may conclude that the method of psychological evaluation used by some of them may not be the most adequate, because the pandemic may not provoke emergence of psychological disorders but, more commonly, psychological symptoms. Therefore, it could be more adequate to use wellbeing and perceived symptoms scales instead of diagnostic scales.

It is also important to highlight that most of the studies are cross-sectional, so the information available only provides a picture of the mental status of people at that point in time. Moreover, many sent their questionnaires in the 1st weeks of implementation of lockdown or social distancing, when respondents had been in isolation for a short time.

## The third quarter effect

The phenomenon known as the “third-quarter effect” was observed in studies in Antarctica and space analogs, but there is no information about it in the experimental and pandemic related studies, which makes it difficult to generalize such findings. The true existence of this phenomenon is still controversial, given that several studies’ results did not confirm its presence.^49^ Bechtel et al.^49^ postulate that this effect may only be a characteristic of finite-time stressful situations. The concept of psychological resilience, i.e., the human capacity to cope successfully under significant adverse conditions^54^ may interestingly be related to the third-quarter effect. In their study in Antarctic environments, Sandal et al.^37^ wrote that resilience may be related to expectations about the duration of the isolation, being associated with the relative passage of time, and that it decreases in the third quarter, when the participants realize that the trip is only half-complete.^55^ Another concept brought out by the authors is “psychological hibernation,” i.e., a reduction in seeking stimulation, or an emotional flatness. This hibernation is associated with stress and may represent a strategy and an adaptive response for coping with the stressors of the extreme conditions.^55^

A retrospective evaluation could be more adequate to assess this phenomenon in a situation in which it is difficult to foresee the exact duration of the isolation (like in pandemics). However, the memory bias generated by stress can influence retrospective data.^56^ Possible options for better evaluation may be through the observations of a trained observer, prospective sequential self-reports, or reports provided by peers and external observers.

Researchers aiming to assess the psychological effects of social isolation should carefully plan and report several contextual characteristics (whenever possible). Figure 3 contains a summary of our recommendations for future research on human isolation, based on this review.

Since there is evidence that crowding is associated with psychological distress,^57^ it is important that the total physical space and the area available per individual are described, in both experimental and naturalistic studies. In the case of naturalistic studies, other environmental conditions such as temperature,^58,59^ seasonality,^60^ and altitude^61^ might influence observed outcomes. Light-dark cycle alterations, including the lack of exposure to daylight and exposure to artificial light at night, have an impact on sleep, mood and other psychological variables^62,63^ and should therefore be described in both experimental and naturalistic studies. In experimental studies, for the assessment of the factors described above, we recommend inclusion of a detailed and complete description of the layout of the isolation setting.

Non-fulfillment of basic needs, including financial assistance and access to basic supplies, increases stress, anxiety, and depression symptoms.^64^ Hence, it is pertinent to report income and/or availability of essential supplies.

As mentioned in the results section, lack of social interactions leads to adverse outcomes, whereas social connectedness improves resilience to adverse situations.^65^ Thus, the possibility of keeping in touch with friends and family during isolation should also be considered when designing and reporting the results. Future studies focusing on the impact of individual *versus* group isolation with different degrees of social contact could contribute to a better understanding of the effects of a person’s social network during isolation.

Maintenance of a daily work routine^66^ and engaging in physical^67^ and leisure activities^68^ can be beneficial for psychological health. In addition, shift work is a major cause of mental health problems, thus indicating a noteworthy confounding factor. For this reason, we suggest that planning and reporting activity and routine characteristics should be included in studies on the effects of isolation.

Based on the information gathered in this review, we suggest that researchers aiming to assess the psychological effects of social isolation in a pandemic situation should precisely characterize the number of days people have been in isolation, the frequency of the isolation (i.e., the amount of time people stayed in isolation), accessibility (i.e., how easy it is to get in and out of the isolation setting), the number of in-person contacts (online and face-to-face), and the participants’ social network. It is important to determine the nature of the setting, collecting information regarding the size of the residence, the number of people living there and their kinship, and some aspects of the environment (windows, open-air spaces). The activities the person does during the period (including work, study, leisure and physical activities), use of protective measures, the amount, quality, and source of the information received about the crisis, as well as an evaluation of the individual risk each participant would be under in the event of becoming infected should also be properly assessed.^11^

## A note on the COVID-19 pandemic

The COVID-19 pandemic notably changed societal views and conceptions of social isolation. Not surprisingly, the scientific community has responded to this social demand and a vast body of evidence was produced from 2020 to 2022. When conducting a third search using the same identifiers in April 2022, a noteworthy increase in the number of records was identified (Supplementary Material S1 and Figure S1, available online-only).

The COVID-related articles identified in the first and second searches (and described in this review) were initial assessments of the “first wave” of the pandemic. An immeasurable number of confounding factors arose with the complex progression of the COVID-19 health issue, contributing to the adverse outcomes related to psychological stress.^69^ Furthermore, social isolation in the context of the COVID-19 pandemic assumed many facets, varying according to the moment of assessment in terms of potential health harms and local public policies. Future studies would greatly contribute to the topic by assessing the impacts of isolation specifically in the years following the spread of COVID-19. However, this was not the focus of the present review. Nonetheless, we believe the recommendations we developed are still adequate for the purpose of studying future situations of isolation in pandemic scenarios.

## Conclusion

The emergence of the COVID-19 pandemic and subsequent lockdowns and social distancing measures adopted worldwide raised questions about the possible health effects of human social isolation. As a result, the number of scientific articles focused on this topic has increased recently and it is likely to keep growing over the next few years. Thus, a systematic literature search was performed in order to review the available evidence on the effects of human social isolation and determine directions for conducting and reporting such studies. Several of the studies included here were not primarily designed to assess isolation itself. Thus, these models are also analyzed in terms of their validity and limitations for the purpose of studying human isolation. Finally, based on the evidence collected here, we designed a group of recommendations for future experimental or naturalistic research on the topic.

Although most studies regarding human social isolation consist of cross-sectional and longitudinal protocols, research performed under experimental and quasi-experimental conditions has provided valuable contributions for determining the causal impact of social connections, and the lack thereof, on health.^6^ In naturalistic conditions, several stress factors can be present, such as harsh or extreme environmental conditions, lack of social contact and fear of infection during an epidemic outbreak. This makes it difficult to separate the effects of isolation itself from other stressors. Experimental models, on the other hand, provide an opportunity to either focus on isolation or to study its relationship with specific stressors (e.g., crowdedness, privacy, or compatibility). However, they might lack external validity because they are performed in extremely controlled conditions.

In spite of confounders and the many differences between models, we were able to observe similarities among outcomes that might be promising research targets for future studies. The data assembled in this review of different contexts, settings, and designs – and the recommendations derived from the analyses – may help us understand the impacts of social isolation on human health, also guiding the design and execution of studies on the topic.

In the context of the current COVID-19 pandemic, social isolation is a central tool to reduce disease transmission. In view of this, we emphasize the necessity of developing measures to foster better compliance with preventive actions and to mitigate the psychological consequences of such measures.^18,70^ From a public health perspective, if isolation is needed, the pressure on public authorities regarding vaccination should be cranked up. Although isolation is notably stressful, the psychological consequences of not adhering to this measure and allowing the virus to spread might be worse.
