# Supplementary material for: Human social isolation and stress: a systematic review of different contexts and recommendations for future studies
Source: Trends Psychiatry Psychother. 2024 Feb 28;46:e20210452. doi: 10.47626/2237-6089-2021-0452 (PMC11332683; doi:10.47626/2237-6089-2021-0452)
Supplement: Supplementary file 1 [file 2238-0019-trends-46-e20210452-suppl.pdf]

Supplemental Material S1

25/05/2020 - 1st search

PUBMED = 363 results
((((social[Title/Abstract]) OR (temporal[Title/Abstract]) OR (environmental[Title/Abstract]) OR (solitary[Title/Abstract])) AND ((isolation[Title/Abstract]) OR (distancing[Title/Abstract]))) OR (confinement[Title/Abstract]) OR (quarantine[Title/Abstract]) OR (seclusion[Title/Abstract]) OR (segregation[Title/Abstract]) OR ((space[Title/Abstract]) AND ((mission[Title/Abstract]) OR (shuttle[Title/Abstract]) OR (analog[Title/Abstract]))) OR (COVID-19[Title/Abstract])) AND ((psychological[Title/Abstract]) AND (stress[Title/Abstract])) AND ((humans) OR (outpatients[Title/Abstract]) OR (astronauts[Title/Abstract]) OR ((offshore[Title/Abstract]) AND (workers[Title/Abstract])) OR (antarctica[Title/Abstract]) OR (antarctic[Title/Abstract]) OR (prisoners[Title/Abstract]) OR (prison[Title/Abstract]))

EMBASE = 551 results
(('social':ab,ti OR 'temporal':ab,ti OR 'environment*':ab,ti OR 'solitary':ab,ti) AND ('isolation':ab,ti OR 'distancing':ab,ti) OR 'confinement':ab,ti OR 'quarantine':ab,ti OR 'seclusion':ab,ti OR 'segregation':ab,ti OR ('space':ab,ti AND ('mission':ab,ti OR 'shuttle':ab,ti OR 'analog':ab,ti)) OR 'covid-19':ab,ti) AND 'psychological':ab,ti AND 'stress':ab,ti AND ('human*' OR 'outpatient*':ab,ti OR 'astronaut*':ab,ti OR ('offshore':ab,ti AND 'worker*':ab,ti) OR 'antarctic*':ab,ti OR 'prisoners':ab,ti OR 'prison':ab,ti)

Scopus = 1185 results
(((TITLE-ABS-KEY (offshore AND worker*)) OR (TITLE-ABS-KEY (human OR outpatient OR astronaut OR antarctic* OR prison*))) AND (((((TITLE-ABS-KEY (social OR temporal OR environment OR solitary)) AND (TITLE-ABS-KEY (isolation OR distancing))) OR (TITLE-ABS-KEY (confinement OR quarantine OR seclusion OR segregation OR covid-19))) OR (TITLE-ABS-KEY (space) AND TITLE-ABS-KEY (mission OR suttle OR analog))) AND (TITLE-ABS-KEY (psychological AND stress)))) AND NOT (ALL (review))

13/08/2020 - 2nd search

PUBMED = 457 results
1st search + “2020”

EMBASE = 717 results
1st search + “2020”

Scopus = 1328 results
1st search + “2020”

07/04/2022 – 3rd search

PUBMED = 57 results
((((social[Title/Abstract]) OR (temporal[Title/Abstract]) OR (environmental[Title/Abstract]) OR (solitary[Title/Abstract])) AND ((isolation[Title/Abstract]) OR (distancing[Title/Abstract]))) OR (confinement[Title/Abstract]) OR (quarantine[Title/Abstract]) OR (seclusion[Title/Abstract]) OR (segregation[Title/Abstract]) OR ((space[Title/Abstract]) AND ((mission[Title/Abstract]) OR (shuttle[Title/Abstract]) OR (analog[Title/Abstract])))) AND ((psychological[Title/Abstract]) AND (stress[Title/Abstract])) AND ((humans) OR (outpatients[Title/Abstract]) OR (astronauts[Title/Abstract]) OR ((offshore[Title/Abstract]) AND (workers[Title/Abstract])) OR (antarctica[Title/Abstract]) OR (antarctic[Title/Abstract]) OR (prisoners[Title/Abstract]) OR (prison[Title/Abstract])) **NOT ((COVID-19[Title/Abstract]) OR (COVID[Title/Abstract]))**

EMBASE = 118 results
(('social':ab,ti OR 'temporal':ab,ti OR 'environment*':ab,ti OR 'solitary':ab,ti) AND ('isolation':ab,ti OR 'distancing':ab,ti) OR 'confinement':ab,ti OR 'quarantine':ab,ti OR 'seclusion':ab,ti OR 'segregation':ab,ti OR ('space':ab,ti AND ('mission':ab,ti OR 'shuttle':ab,ti OR 'analog':ab,ti))) AND 'psychological':ab,ti AND 'stress':ab,ti AND ('human*' OR 'outpatient*':ab,ti OR 'astronaut*':ab,ti OR ('offshore':ab,ti AND 'worker*':ab,ti) OR 'antarctic*':ab,ti OR 'prisoners':ab,ti OR 'prison':ab,ti) **NOT 'covid-19':ab,ti**

Scopus = 37 results
(((TITLE-ABS-KEY (offshore AND worker*)) OR (TITLE-ABS-KEY (human OR outpatient OR astronaut OR antarctic* OR prison*))) AND (((((TITLE-ABS-KEY (social OR temporal OR environment OR solitary)) AND (TITLE-ABS-KEY (isolation OR distancing))) OR (TITLE-ABS-KEY (confinement OR quarantine OR seclusion OR segregation))) OR (TITLE-ABS-KEY (space) AND TITLE-ABS-KEY (mission OR suttle OR analog))) AND (TITLE-ABS-KEY (psychological AND stress)))) AND NOT (ALL (review)) **AND NOT (ALL (COVID-19))**

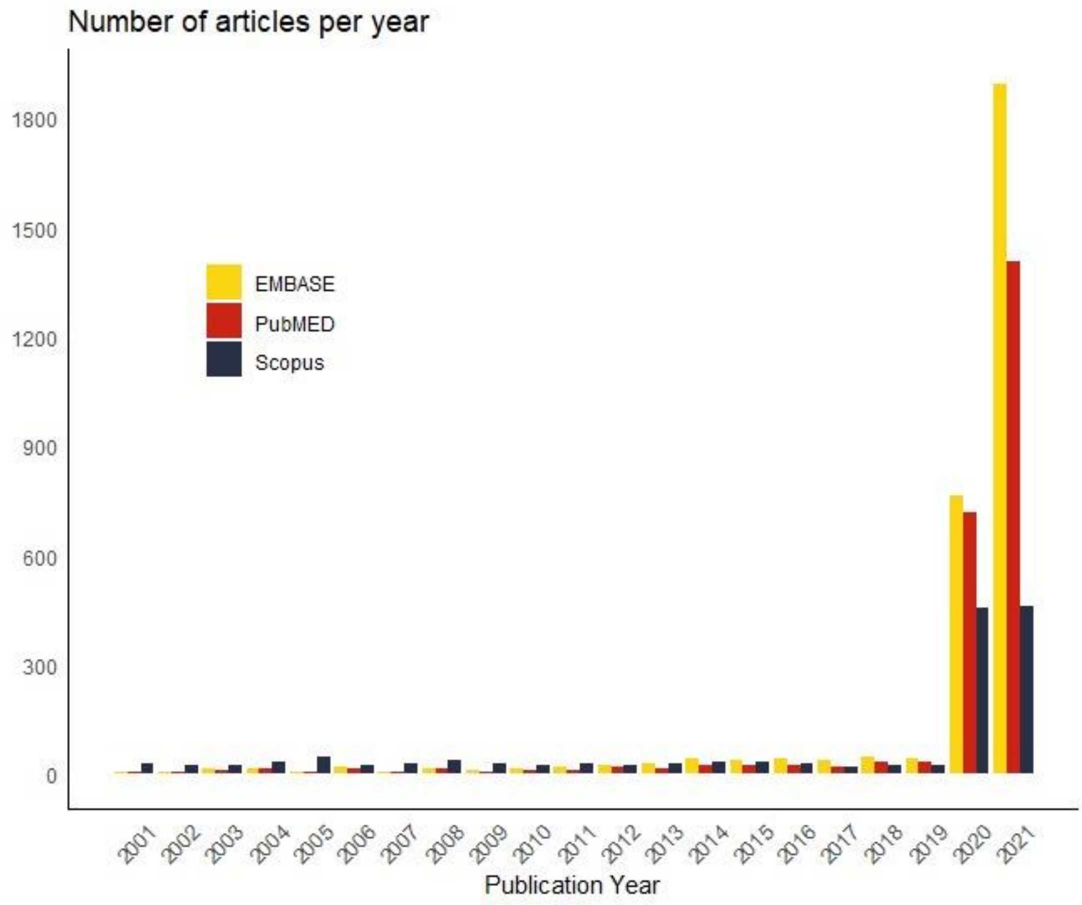

Figure S1 - Number of articles retrieved per year using search terms.

Table S1 - Main outcomes related to social isolation separated by categories

Reference	Model	Assessment (instrument)	Outcomes						
			**Stress/distress/ PTSD**	**Affective**	**Cognitive**	**Anxiety**	**Somatic/sleep**	**Conflicts/ sociability**	**Objective measures**
Hawryluck et al., 2004[1]	Pandemics	Stress/distress (IES-R); Depression (CES-D)	Duration of quarantine influence PTSD symptons and correlate with other symptons of poor mental health. Lower household income and acquaintance with or exposure to someone with SARS were associated with higher stress symptoms.	CES-D score >16 in 31.2% of quarantined persons. Depressive symptoms associate with duration of quarantine. Acquaintance with or exposure to someone with SARS was associated with higher depressive symptoms.	-	Inadequate information about the disease brought feelings like anger, frustration, disappointment and anxiety.	-	-	-
Reynolds et al., 2008[2]	Pandemics	Stress/distress (IES-R)	Stress associated with longer duration of quarantine and compliance with quarantine.	-	-	-	-	Impossibility of visiting family and friends and the fear of infecting themselves or others rated as the most difficult social facts about quarantine.	-
Yuan et al., 2020[3]	Pandemics	Stress/distress (SRQ); Sleep (PSQI)	Emotional state mostly unchanged during the two-week study period.	-	-	Medical staff, busines managers and officials were more affected by anxiety than the rest of the participants.	36.43% of the participants reported severely impaired sleep quality (mainly young adults).	-	-
Meo et al., 2020[4]	Pandemics	Psychological wellbeing and learning behaviour questionaire	-	About 25% of the participants felt depressed during the quarantine period of two weeks.	Quarantine negatively affected the concentration, emotional response and work performance.	-	-	-	-
Gonçalves et al., 2020[5]	Pandemics	Well-being (WHO-5); Affect (UCLA); Stress/distress (PSS-10); General Health	Number of online interactions negatively correlated with stress; in-person contact showed a positive	Depressive symptons were associated with isolation.	-	Concern with the situation of COVID-19 in Brazil relates to anxiousness.	-	Satisfaction with the quality and number of online social interactions associated	-

		Questionnaire (GHQ-12); Anxiety (GAD-7); Depression (CES-D).	connection with stress. No relationship with days of isolation. Risk groups associated with adverse psychological symptoms, and negatively associated with well-being.					positively with well-being and negatively with stress.	
Shi et al., 2020[6]	Pandemics	Depression (PHQ-9); Stress/distress (ASDS); Anxiety (GAD-7); Sleep (ISI).	Prevalence of stress of 24,4% (total sample), 27,3% (frontline workers) and 42,4% (family members or friends of patients with COVID-19). Being younger than 40 years, male, unmarried, having lower income, and a personal history of psychiatric disorders associated with the depression, anxiety, insomnia and stress.	Symptons of depression present in 27.9% of respondents, 30,4% for frontline workers and 46,7% for family members or friends of patients with COVID-19.	-	Symptoms of anxiety in 31.6% (total sample), 34% (frontline workers) and 49,3% (family members or friends of patients with COVID-19).	Prevalence of insomnia of 29.2% (total sample), 34% (frontline workers) and 48,4% (family members or friends of patients with COVID-19).	-	-
Rey et al., 2020[7]	Pandemics	Stress/distress (IES-R); Depression, Anxiety, and Stres (DASS-21)	22.4% report moderate to severe stress; higher distress associated with number of days without leaving their home, and higher the time spent getting informed, being exposed to someone diagnosed COVID- 19 or having COVID-19 symptoms.	11.4% showed mild; 14.8% moderate; 6.3% severe and 8.5% extremely severe depression; students and unemployed respondents showed higher levels .	-	5.3% showed mild; 11.3% moderate; 4.8% severe and 9.2% extremely severe anxiety.	-	-	-
Chen et al., 2020[8]	Pandemics	Mental status (mental and behavioral questionnaire); Behavioral patterns (BRQ)	Bad behavior during isolation such as drinking and smoking leads to more serious psychological problems.	63% of participants classified as medium-risk for mental health problems; male and younger students were more depressed.	-	Psychological problems related to excessively negative pandemic information.	-	-	-
Peng et al., 2020[9]	Pandemics	Depression (ZSRDS); Anxiety (ZSRAS); Stress/distress (IES-R); Sleep (PSQI)	-	Depressive symptons in 6.21% of the participants. Being young, less educated, single and having other	-	-	-	-	-

				mental health symptons were risk factors for symptons of depression.					
Elmer et al., 2020.[10]	Pandemics	Depression (German version of the CES-D); Anxiety (GAD-7); Stress/distress (PSS); Affect (UCLA); Sociability (German version of the BFI).	Overall higher stress after COVID-19 breakdown; students with more worries and female students are more likely to become stressed.	More feelings of loneliness, and depressed mood since the COVID-19 breakdown; having more worries and personal problems, living alone and being female associated with higher risk for depression. Students with more emotional support were less depressed.	-	Higher anxiety since COVID-19 breakdown; participants with a smaller social network, isolation in student network, more worries, less social interactions and female sex were more likely to become anxious.	-	Fewer interactions with partners and fewer study partners; higher feelings of isolation.	-
Yi et al., 2014[11]	Space	Affect (VAS, POMS-SF)	-	No differences in mood disturbance, feeling stressed, unhappy, mentally fatigued, or tired.	-	-	Increased subjective feelings of sickness compared to baseline (not corroborated by medical records).	-	Increased salivary cortisol levels from the 360th day onwards.
Luger et al. 2014[12]	Space	Affect (DES and EER), Stress-coping strategies (SVF), Current stress (KAB), Sleeping and dreaming questionnaire, sleepiness (ESS and SSS)	-	No changes in emotional status.	-	-	Increased sleep duration, bed rest time, and frequency of wake-ups from 4 weeks of isolation onwards.	Poorer social competence and teamwork in 'control' group in the first two weeks	-
Yuan et al., 2019[13]	Space	Recovery-Stress (RESTQ), Affect (PANAS), Isolated and Confined Environments Questionnaire (ICE-Q), Cortisol levels (blood)	No changes in total stress and total recovery integrative variables	Total adaptation increased in the first month and remained elevated. Decrease in hostility and negative emotions; increase in emotional adaptation and occurrence of collateral acts in the second half of confinement (4th, 5th and 6th months).	-	No changes in anxiety intensity.	Less frequency of exercises after the 4th month. Behavioral and social activities were lower in the 2nd month.	Conflicts/pressure tended to decrease throughout confinement.	Cortisol increased in 1st month, but levels normalized in the following months.

Basner et al., 2014[14]	Space	Affect (VAS, SDS and POMS-SF), Depression (BDI-II), Conflict (CQ)	No changes in psychological distress.	No changes in depression or total mood disturbance.	-	-	No changes in sleep quality. Greater feelings of sickness, tiredness, fatigue and loss of pleasure in the second half of the mission. Vigor and activity were lowest in mission quarter 2.	Crew-reported conflicts with mission control were reported 5 times more often than conflicts among crew members.	-
Kanas et al., 2001[15]	Space	Affect (POMS), Sociability (GES)	-	Crewmembers presented less dysphoria, expressiveness, independence, anger, aggression, and innovation than the controls.	-	-	-	-	-
Rosnet et al., 1998[16]	Space	Anxiety (STAI); Videotaped interpersonal distance measure	-	-	-	No significant variation.	-	-	-
Sandal et al., 2003[17]	Submarine	Stress (UHI and Submarine Stress Questionnaire), Sleep (Submarine Stress Questionnaire), Affect (UHI), Anxiety (UHI), Depression (UHI)	Increased homesickness and stress related to social factors, and feeling isolated.	-	-	-	Health complaints were higher in crew members on the 10-day mission compared to the 40-day mission	-	Increase in the cortisone values of the submariners
Décamps et al., 2005[18]	Antarctica	Stress/Distress, Affect, Physical symptoms, Cognitive and Social (59-item observation grid by the mission's doctor)	Stress reactions decreased. around the third-quarter (weeks 31-40).	Increase in mood variations during the second half of the isolation period.	No significant variation of the number of occupational reactions.	-	Significant decrease of somatic reactions before the third quarter; then remained stable.	Social reactions decreased in the middle of isolation (weeks 31-40) and increased in the remaining periods.	-
Peri et al., 2000[19]	Antarctica	Affect (POMS); Stress/distress (WCI)	Reduced search for social support and decrease in problem-focused coping.	No significant differences.	-	-	-	-	-
Sandal et	Antarctica	Coping (UCL); Sleep	All observed coping	Positive affects and	-	-	Subjective sleep	-	-

al., 2018[20]		(sleep diary); Affect (PANAS)	strategies decreased during midwinter.	coping strategies scores decreased around cold and dark mid-winter months.			quality decreased during the stay.		
Strewe et al., 2019[21]	Antarctica	Stress/distress (CST, PTSS-10); Anxiety (STAI); Objective measurements (cortisol in saliva; urine catecholamines; seric endocannabinoid, N-acylethanolamide, recall antigen- and mitogen-stimulated cytokine profiles and cytokine production)	Stress level was low during the whole year, not differing according to time of data collection (morning/evening). Small but not significant increase in negative feelings.	-	-	Low anxiety throughout the entire observation period.	-	-	Higher salivary cortisol in females in some months. Norepinephrine excretion during the day was higher in the first months and dropped at the onset of the Antarctic winter. Consistently elevated endocannabinoids and N-acylethanolamides.
Caputo et al., 2020[22]	Antarctica	Affect (PANAS); Gene expression profile; Anxiety (ECR)	-	Participants with insecure attachment style reported stronger negative mood compared with secure attachment style.	-	No influences in anxiety.	-	-	Prolonged confinement and isolation altered overall patterns of gene expression. Reduced alteration in individuals characterised by a secure attachment style.
Shimamiya et al., 2004[23]	Experimental/ lab conditions	Affect (Face scale test); Objective measurements (leukocyte subpopulations, natural killer cells, CD69-expressing lymphocytes in blood samples)	-	Lower mood scores at the beginning, that improved towards the end of the experiment.	-	-	-		Changes in Immune markers associated with the confined environment.

Smith et al., 1972[24]	Experimental/ lab conditions	Stress/distress (SSS); Hostility (Hostility Scale); Anxiety (STAI)	Low stress during confinement (but significantly higher than baseline). Stress peaked at day 5 and reached its lower level on day 13 (around third-quarter). Lowest stress in the least crowded three-man groups; highest stress in the crowded threeman groups. Less stress in three-man groups than in dyads.	-	-	Increased state anxiety, higher in the two-man-group. Combining leadership and group size, triads led by senior men registered the lowest state anxiety.	-	Groups led by junior leaders display more hostility. Incompatibility between members of the group led to significantly higher hostility; greater hostility toward partners in less crowded groups.	-
Zubek et al., 1969[25]	Experimental/ lab conditions	Stress/distress (SSS); Objective measurements (EEG); Cognitive (MPQ and Intellectual battery); Anxiety (MPQ); Physical symptoms (MPQ)	No difference between the confined, the socially isolated and the ambulatory control groups regarding stress levels.Quitting rates (1 in CF; 5 in SI) might result from stress in confinement and social isolation situations.	-	Social isolation group showed worse performance on intellectual battery. Subjective phenomena (experiences of a hallucinatory-like nature, inefficient thought processes, reminiscence and vivid memories, and changes in body image and self-appraisal) associated with confinement.	Both the social isolation and the confinement groups reported a greater incidence of worry than the control group.	Subjective restlessness and restless acts were associated solely with confinement, not increasing with social isolation.	-	Decrease in EEG frequency in both of the experimental groups. 14 of the I5 subjects in each of the two groups exhibited a lower EEG frequency on the postexperimental relative to the preexperimental test period.
Taylor et al., 1968[26]	Experimental/ lab conditions	Stress/distress (SSS); Anxiety (general behavioral disposition for anxiety proncness)	The expectancy of a longer isolation period generates more stress. Long mission in separate compartments with no outside contact proved to be the most stressful.	-	-	No effect on anxiety levels. The 4-day groups maintained stable and significantly lower level of anxiety state than the 20-day groups.	-	-	-
Zuckerman et al., 1966[27]	Experimental/ lab conditions	Stress/distress (MPQ) Depression (MAACL); Anxiety (MAACL; MPQ);	Stress was higher after the first sessions of perceptual and social isolation than	-	Loss of reality contact, hallucinations, dreams, and	Anxiety was significantly greater in the isolation than in	-	-	No significant effects for occasion of testing were obtained for any of

		Cognitive (MPQ); Objective measurements (Heart rate, basal skin conductance, breathing rate and amplitude. Blood ACTH; urine LH, 17-KGS, and 17-KS; plasma TSH, hydrocortisone, corticosterone, PBI and BEI).	after the second ones. Perceptual restriction produce stress response beyond that produced by the nonperceptually restricted confinement situation.		body-image changes were significantly higher in the isolation condition than in the stimulation condition.	the stimulation condition.			the hormones measured.

Supplemental Material S2

Main results

1. Experimental and quasi-experimental models

The samples of the five experimental and quasi-experimental studies were composed only by males. In three of them subjects were young (under 30 years of age) and in the other two, the age was not described. Duration of isolation ranged from two 8-hour sessions to 21 days.

Stress was assessed in four of the five experimental and quasi-experimental studies included in this review using either the Standard Stress Scale or the Multidimensional Personality Questionnaire[21,24–27]. In a study evaluating the effects of compatibility, crowding, group size and leadership seniority on several outcomes, groups composed of three men usually reported less stress than two-men groups. When in a crowded condition, however, three-men groups indicated feeling the most stressed[24]. Another study investigated the effects of privacy, expectations about length of social isolation and degree of contact with the outside world in socially isolated groups and found that the expectation of a longer isolation period generated more stress and led to higher quitting rates. Groups expecting longer isolation with more privacy and no outside contact reported the greater stress, while groups expecting shorter missions with outside stimulation and privacy were the least stressed. In this same study, more than half of the participants did not complete the protocol, 67% of which expected a longer isolation period[26]. Another study with a 1-week duration showed no statistical difference regarding stress levels when comparing groups that were confined but allowed to have social contact, confined and socially isolated and controls. Quitting rates, nonetheless, were higher in the socially isolated group[25].

Anxiety was also investigated in four of the five articles included. When comparing the effects of crowding, group size and leadership seniority on this outcome, two-men-group subjects registered significantly higher state anxiety throughout confinement than those in three-men groups; moreover, triads led by senior men registered the lowest state anxiety[24]. In another study, confined groups reported greater incidence of worry than the control group[25]. When subjects had different expectations about the length of isolation, groups expecting shorter periods maintained a fairly stable and significantly lower level of anxiety state throughout confinement; the expectation of longer isolation under conditions of privacy produced the greatest levels of anxiety, with gradual increases over days; groups expecting longer isolation periods with privacy and without outside contact showed a significantly sharp increase of anxiety after five days of protocol, when all individuals in this condition quitted[26]. In a study in which subjects spent two sessions of eight hours confined and in social isolation, either with or without sensory deprivation (visual and auditory restriction achieved through the use of blacked-out goggles and acoustical earmuffs), anxiety was greater in the sensory deprivation condition[27].

Several objective outcomes were assessed. A 10-day confinement period produced changes in the distribution of leukocyte subpopulations, natural killer (NK) cells and CD69-expressing lymphocytes in confined subjects[23]. Alterations in occipital alpha frequency measured by electroencephalography were observed after one week of confinement[25]. When subjects were confined and socially isolated for eight hours, breathing rate was lower in the group under sensory deprivation, but no differences were found in other physiological measurements, such as heart rate, basal skin conductance, fluctuations in skin conductance and breathing amplitude. Endocrine responses were also measured, and some hormones (17-KGS, LH, 17-KS and TSH) were altered by the sensory deprivation condition[27], although these where punctual measurements and we are unable to infer the rhythmicity of synthesis or secretion of these hormones.

Two of the articles assessed cognitive outcomes and showed that the social isolation group had the worst performance in an intellectual battery (consisting of tests measuring numerical reasoning, number facility, abstract reasoning, verbal reasoning, verbal fluency, space relations, digit span, recall, recognition, cancellation of numbers, and dexterity) and loss of contact with reality. Subjective phenomena (i.e. experiences of a hallucinatory-like nature, inefficient thought processes, reminiscence and vivid memories and changes in body image and self-appraisal) resulted from confinement, despite

social contact or lack thereof[25]. In another study, participants placed in the sensory deprivation condition during an eight-hour confinement and social isolation scored significantly higher on the questionnaire measuring loss of reality contact, hallucinations, dreams, and body-image changes[27].

Two studies assessed affective outcomes. Men confined for 10 days in groups of five had their mood assessed through the face scale test, and the results showed that the subjects' moods were low at the beginning and then improved toward the end of the experiment[23]. When depression symptoms were evaluated, they were found to be higher in participants subjected to sensory deprivation[27]. A higher incidence of subjective restlessness and restless acts was observed in groups confined for 7 days than in non-confined ambulatory controls, regardless of social isolation[25].

The effects of compatibility, crowding, group size and leadership seniority on hostility showed that groups led by junior leaders tended to display more hostility. Also, incompatibility between members of the group led to significantly higher hostility and greater hostility toward partners was revealed by subjects in less crowded groups than by crowded subjects[24].

2. Space mission and analogues

All data collected from five space analogues and an official space flight reflect homogenous samples of mostly males under 40 years of age. These studies follow a longitudinal design, with the duration of isolation ranging from a few months to 520 days. Overall, no significant difference was reported in scores of perceived stress, anxiety, or mood disturbances. However, one controlled study observed that crewmembers presented less dysphoria, expressiveness, independence, anger and aggression compared to mission control personnel[15].

One study reported increased subjective feelings of sickness in the assessments of days 360 and 510 compared to baseline[11]. This study also reported significantly higher salivary cortisol levels from the 360th day onwards. Another study observed significant differences on the second half of the mission (4th, 5th and 6th months) compared to the first[13]. Thus, from the 4th month of isolation on, subjects exercised less, increased the masseter tone by 6–14% and showed an increase in collateral acts (a roll of psychosomatic events that might indicate stress and anxiety behavior) assessed by Ethological Monitoring. Moreover, cortisol levels increased in the first month, but were normalized in the following months. Similarly, Basner and colleagues[14] described greater feelings of sickness, tiredness, fatigue and loss of pleasure in the second half of the mission, while vigor and activity were lowest in the second mission quarter, although no changes in sleep quality were reported. Nonetheless, Luger and colleagues[12] reported increased sleep duration, bed rest time and frequency of wake-ups from 4 weeks of isolation onwards. Moreover, compared to mission control personnel, Luger found poorer social competence and teamwork in isolated individuals in the first two weeks[12]. However, Basner reported five times less conflicts in crew members[14]. Finally, Yuan described that conflicts and pressure tended to decrease throughout confinement, while behavioral and social activities were lower in the 2nd month.

3. Submarine mission

Only one study conducted in a submarine setting was retrieved[17], in which one sample was recruited for a 40-day mission and another, for a 10-day mission, while also recruiting office workers and a control group of military recruits. One of the purposes of this study was to observe distinct personality traits and coping strategies in this homogenous sample of men (only one woman was recruited). Submariners reported low frequency of health complaints in all measurements and these scores were even lower than the control group at baseline. In addition, no significant differences between scores in the first and last weeks of the mission for any of the crews were found. However, the authors recorded an increase in cortisone values of the submariners, which also reported increased homesickness and stress related to social factors and feeling isolated after the missions compared with baseline. Cortisone levels and health complaints were higher in crew members on the 10-day than in the 40-day mission.

4. Antarctic

The five Antarctic studies are composed of similar samples of mostly males under 60 years of age. These studies follow a cohort design, with the duration of isolation ranging from two months to one year.

Stress-related outcomes were evaluated in four out of the five Antarctic studies included in this review. A longitudinal assessment of psychological adaptation during a winter-over in Antarctica showed that stress reactions decreased around the third-quarter of the stay, contradicting the “third-quarter phenomenon” - the idea that this period is when most discomfort is reported[18]. A study conducted during the summer months investigated the coping strategies used by participants to deal with the Antarctic environment and maintain emotional stability. At the end of the stay there was a significant decrease in coping strategies based on seeking social support and problem analysis and solution; coping by acceptance and attempting to get something positive out of the situation increased, though not significantly[19]. Another investigation on stress coping strategies, performed during the winter months, showed that all observed coping strategies (active problem solving, palliative reactions, avoidance and passive expectancies, and comforting cognitions) decreased during midwinter, the darkest and coldest period, which also corresponded to the third-quarter of the expedition[20]. A study about sex differences in stress responses during confinement in Antarctica showed that stress levels remained low during the whole year, not differing between sexes or according to time of data collection. When completing the Post-Traumatic Stress Scale (applied 2 months before, in the middle and 3-4 months after the expedition), participants from both sexes reported no increased feelings of anxiety when talking about the prior month, but exhibited a small, but not significant, increase in negative feelings during the past few days at all time points[21].

Affective outcomes were also assessed in four of the five Antarctic studies. Participants spending 50 weeks in Antarctica showed stable reports of pessimistic feelings, sadness, and lack of motivation before the middle of the isolation period, which then significantly increased until the end of the stay[18]. During a summer campaign of 2-5 months subjects presented no significant differences in mood states between baseline and end of the isolation period. However, a small increase of confusion and fatigue, and a small reduction of vigor were observed[19]. Another study of winter-over stay in Antarctica showed that negative affective states (e.g. feeling afraid, upset, ashamed) were continuously low during the entire isolation period, while positive affective states (e.g. feeling excited, inspired, alert) were least reported during the mid-winter months[20]. In a study evaluating the association between attachment style and resilience in extreme environments, participants with insecure attachment style reported stronger negative mood in comparison to participants with secure attachment style throughout the entire mission. Positive mood was stable among individuals with both secure and insecure attachment styles throughout the isolation period[22].

In regard to physical symptoms and sleep, one winter-over study showed significant decrease of somatic reactions (e.g. insomnia, appetite alterations, headaches) until the middle of the isolation period, after which the number of somatic reactions stabilized[18]. In another study, participants completed a sleep diary during the first week of every month for ten months and the results indicated a negative trend over time in subjective sleep quality, with a slight increase at the end of the stay[20].

Anxiety was evaluated in two winter-over Antarctic studies. Strewe and colleagues reported that state and trait anxiety scores were low throughout the entire period and no differences between sexes were observed[21]. Caputo and colleagues found that prolonged isolation and confinement did not influence individual anxiety, whereas state anxiety was constant throughout the mission. Similarly, attachment style did not influence absolute anxiety nor its progression during the isolation period[22].

The study aiming to identify sex differences in stress and immune responses during confinement also included objective measurements. Salivary cortisol concentrations in the morning were significantly higher in females than males in April, May and July, and in both sexes the cortisol ratio between morning and evening values was higher throughout the expedition than at baseline. No sex differences were observed in relation to cytokine profiles after *in vitro* stimulation, but significant time-dependent changes were noticed: the concentration of IFN-γ rose continuously and peaked around the middle of the expedition (but showed significant changes to baseline data collection only for males), then declined gradually to reach baseline levels at post data collection (PDC); both sexes showed significant increases

in IL-10 concentrations at PDC; IL-2 concentrations peaked in June (but did not reach statistical significance in comparison to baseline values) and TNF concentrations declined during the expedition in comparison to baseline. Blood cell counts indicated that hemoglobin increased significantly during the whole isolation period compared to baseline and hematocrit values were significantly higher than baseline in the months of March, July, September and October in females. Significant differences in thrombocyte concentration in comparison to baseline were found in females in June and July and for males in March and July. Leukocytes and granulocyte levels were higher during confinement with a dip in winter whereas lymphocytes were significantly elevated throughout the entire isolation period[21]. The study evaluating the association between attachment style and genomic and physiological resilience in extreme environments showed that participants with a secure attachment style exhibited significantly lower salivary cortisol concentrations compared with participants with insecure attachment style one month after arriving at the station. Prolonged exposure to confinement and isolation induced significantly different gene expression profiles in all subjects, deregulating genes related to mitochondrial function, protein synthesis, immune response and circadian rhythms. Individuals with insecure attachment style, however, showed a higher proportion of differentially expressed genes than participants with secure attachment style[22].

Décamps and Rosnet assessed occupational and social reactions in participants during a winter-over in Antarctica and found that, while no significant variation in the number of occupational reactions (e.g. overinvestment in work, overestimation of workload, inability to complete any task) were observed throughout the stay, social reactions (e.g. withdrawal within oneself, criticism, distrust) generally increased significantly during the stay, with a significant decrease following the middle of the isolation period[18].

5. Pandemics

The pandemic requires us to look for effective preventive strategies, like social distancing, which necessarily brings individuals into isolation. In the reviewed studies, the mental health variables most commonly assessed are stress, depression and anxiety, although a few articles found significant results in other psychological aspects, like boredom, subjective isolation[2], insomnia[6], and general worries[7]. In the pandemic's model, the duration of isolation seems to be an important predictor of psychological impact[1,2,7], although this factor was not assessed in all studies.

The association between demographic variables and the presence of psychological symptoms varied among studies. One study[6] showed that being under 40 years old, having a history of psychiatric disorders, being male and being unmarried were associated with symptoms of depression, anxiety, insomnia, and acute stress. In another article[7], women and *people who were in a relationship but not cohabitating* showed higher psychological impairment, and students and unemployed respondents showed higher levels for depression. The same article found that participants aged 18–24, followed by 25-34 showed the highest psychological impact. It also states that participants with the highest levels of psychological impact, stress, and anxiety were students, and the least affected were retired people. Another study[9] found that the depressed group was younger, less educated and more likely to be unmarried. Between the student population, one article found that women were more likely to become depressed, anxious, stressed and lonely[10], while another found that male and younger students were more prone to depression[8].

Being a frontline worker is associated with elevated risk for insomnia and acute stress symptoms[6]. Being a healthcare worker is also associated with higher stress level[2]. Not surprisingly, being part of the high-risk population in case of COVID-19 infection is associated with higher levels of psychological impact, including anxiety, stress and depressive symptoms[7] and is negatively associated with well-being[5]. Participants with confirmed or suspected disease, or in proximity with people infected with SARS-CoV-2 or who tested positive for COVID-19 had a tendency towards greater psychological impact[1,6,7]. Moreover, one study found that in-person contact face-to-face, in general, had a positive connection with stress[7].

The satisfaction with the amount and the quality of information received about the current situation appears to be very important to psychological well-being. In one study[7], 44.2% of the respondents indicated that they needed more information, and these participants showed poorer mental

health. Similar results were found in other articles[2]. Along this line, one interesting result is that participants who spent three or more hours getting informed about the pandemic showed higher psychological impact, anxiety and depression compared to people who spent less time on this task[7].

The household income has an influence on the mental status of people, given that lower family incomes were associated with higher psychological impact[1,2,6,7]. Only one study[7] made correlations between residence size, family composition and psychological aspects of people. It found that participants with houses sized more than 120 square meters showed lower psychological impact, stress, anxiety and depression. Also, respondents with an open-air space in the house showed slightly lower psychological impact. It searched, as well, for an association between dwellers density, which authors computed as an “overcrowding index” (calculated based on the residence size and the household size) and levels of distress. Participants living in a household with a low overcrowding index showed lower distress.

The impact of social isolation on students appears to be of great interest to researchers given that 3 out of 10 studies selected are exclusively directed towards this population. In one study[4], about one fourth of the students reported feeling depressed during the quarantine period of two weeks, and some of them referred to having symptoms of cognitive impairment, e.g., “deterioration in work performance and studying subjects contents”, “difficulty concentrating on the studies”. Another study[8] states that behaviors, such as drinking and smoking, and “receiving too much negative pandemic information” are associated with more serious psychological problems. It also shows that academic stress has a negative impact on depression. The third study[10] is a cohort study, which reported that, in general, students became more depressed, slightly more anxious, more stressed and more lonely since the crisis. It also shows that having smaller personal networks, living alone and having less interactions with other individuals may lead to higher levels of, respectively, loneliness, depression and anxiety. On the contrary, students with more emotional support were less depressed and less lonely.

The studies identified some protective factors against the psychological impact of social distancing. Physical and leisure activities, mainly watching films or shows, were associated with lower stress, anxiety and depression scores[7]. Likewise, reading and handicraft or art activities were related to lower scores in all psychological dimensions assessed[7]. Having work activities, excluding frontline and healthcare workers, was associated with lower risk for symptoms of depression, anxiety and insomnia[6].

REFERENCE LIST

1. Hawryluck L, Gold WL, Robinson S, Pogorski S, Galea S, Styra R. SARS control and psychological effects of quarantine, Toronto, Canada. *Emerg Infect Dis*. 2004;10(7):1206-1212. doi:10.3201/eid1007.030703
2. Reynolds DL, Garay JR, Deamond SL, Moran MK, Gold W, Styra R. Understanding, compliance and psychological impact of the SARS quarantine experience. *Epidemiol Infect*. 2008;136(7):997-1007. doi:10.1017/S0950268807009156
3. Yuan S, Liao Z, Huang H, et al. Comparison of the Indicators of Psychological Stress in the Population of Hubei Province and Non-Endemic Provinces in China During Two Weeks During the Coronavirus Disease 2019 (COVID-19) Outbreak in February 2020. *Med Sci Monit Int Med J Exp Clin Res*. 2020;26:e923767. doi:10.12659/MSM.923767
4. Meo S.A., Abukhalaf A.A., Alomar A.A., Sattar K., Klonoff D.C. Covid-19 pandemic: Impact of quarantine on medical students’ mental wellbeing and learning behaviors. *Pak J Med Sci*. 2020;36(COVID19-S4):S43-S48. doi:10.12669/pjms.36.COVID19-S4.2809
5. Gonçalves A.P., Zuanazzi A.C., Salvador A.P., Jaloto A., Pianowski G., Carvalho L.D.F. Preliminary findings on the associations between mental health indicators and social isolation during the COVID-19 pandemic. *Arch Psychiatry Psychother*. 2020;22(2):10-19. doi:10.12740/APP/122576
6. Shi L, Lu ZA, Que JY, et al. Prevalence of and Risk Factors Associated With Mental Health Symptoms Among the General Population in China During the Coronavirus Disease 2019 Pandemic. *JAMA Netw Open*. 2020;3(7):e2014053. doi:10.1001/jamanetworkopen.2020.14053
7. Rodríguez-Rey R, Garrido-Hernansaiz H, Collado S. Psychological Impact and Associated Factors During the Initial Stage of the Coronavirus (COVID-19) Pandemic Among the General Population in Spain. *Front Psychol*. 2020;11:1540. doi:10.3389/fpsyg.2020.01540

8. Chen B, Sun J, Feng Y. How Have COVID-19 Isolation Policies Affected Young People's Mental Health? - Evidence From Chinese College Students. *Front Psychol*. 2020;11:1529. doi:10.3389/fpsyg.2020.01529
9. Peng M., Mo B., Liu Y., et al. Prevalence, risk factors and clinical correlates of depression in quarantined population during the COVID-19 outbreak. *J Affect Disord*. 2020;275((Peng M.; Song X.; Liu L.) Department of Psychiatry, Shenzhen Nanshan People's Hospital of Shenzhen University, Shenzhen, China):119-124. doi:10.1016/j.jad.2020.06.035
10. Elmer T, Mepham K, Stadtfeld C. Students under lockdown: Comparisons of students' social networks and mental health before and during the COVID-19 crisis in Switzerland. *PloS One*. 2020;15(7):e0236337. doi:10.1371/journal.pone.0236337
11. Yi B, Rykova M, Feuerecker M, et al. 520-d Isolation and confinement simulating a flight to Mars reveals heightened immune responses and alterations of leukocyte phenotype. *Brain Behav Immun*. 2014;40:203-210. doi:10.1016/j.bbi.2014.03.018
12. Luger TJ, Stadler A, Gorur P, et al. Medical preparedness, incidents, and group dynamics during the analog MARS2013 mission. *Astrobiology*. 2014;14(5):438-450. doi:10.1089/ast.2013.1128
13. Yuan M, Custaud MA, Xu Z, et al. Multi-System Adaptation to Confinement During the 180-Day Controlled Ecological Life Support System (CELSS) Experiment. *Front Physiol*. 2019;10:575. doi:10.3389/fphys.2019.00575
14. Basner M, Dinges DF, Mollicone DJ, et al. Psychological and behavioral changes during confinement in a 520-day simulated interplanetary mission to mars. *PloS One*. 2014;9(3):e93298. doi:10.1371/journal.pone.0093298
15. Kanas N, Salnitskiy V, Grund EM, et al. Psychosocial issues in space: results from Shuttle/Mir. *Gravitational Space Biol Bull Publ Am Soc Gravitational Space Biol*. 2001;14(2):35-45.
16. Rosnet E, Cazes G, Vinokhodova A. Study of the psychological adaptation of the crew during a 135 days space simulation. *Acta Astronaut*. 1998;42(1-8):265-272. doi:10.1016/s0094-5765(98)00123-4
17. Sandal GM, Endresen IM, Vaernes R, Ursin H. Personality and coping strategies during submarine missions. *Hum Perform Extreme Environ J Soc Hum Perform Extreme Environ*. 2003;7(1):29-42.
18. Décamps G, Rosnet E. A longitudinal assessment of psychological adaptation during a winter-over in Antarctica. *Environ Behav*. 2005;37(3):418-435. doi:10.1177/0013916504272561
19. Peri A, Scarlata C, Barbarito M. Preliminary studies on the psychological adjustment in the Italian Antarctic summer campaigns. *Environ Behav*. 2000;32(1):72-83. doi:10.1177/00139160021972432
20. Sandal GM, van deVijver FJR, Smith N. Psychological Hibernation in Antarctica. *Front Psychol*. 2018;9:2235. doi:10.3389/fpsyg.2018.02235
21. Strewe C, Moser D, Buchheim JI, et al. Sex differences in stress and immune responses during confinement in Antarctica. *Biol Sex Differ*. 2019;10(1):20. doi:10.1186/s13293-019-0231-0
22. Caputo V, Pacilli MG, Arisi I, et al. Genomic and physiological resilience in extreme environments are associated with a secure attachment style. *Transl Psychiatry*. 2020;10(1):185. doi:10.1038/s41398-020-00869-4
23. Shimamiya T, Terada N, Hiejima Y, Wakabayashi S, Kasai H, Mohri M. Effects of 10-day confinement on the immune system and psychological aspects in humans. *J Appl Physiol Bethesda Md 1985*. 2004;97(3):920-924. doi:10.1152/japplphysiol.00043.2004
24. Smith S, Haythorn WW. Effects of compatibility, crowding, group size, and leadership seniority on stress, anxiety, hostility, and annoyance in isolated groups. *J Pers Soc Psychol*. 1972;22(1):67-79. doi:10.1037/h0032392
25. Zubek JP, Bayer L, Shephard JM. Relative effects of prolonged social isolation and confinement: Behavioral and EEG changes. *J Abnorm Psychol*. 1969;74(5):625-631. doi:10.1037/h0028053
26. TAYLOR DA, WHEELER L, ALTMAN I. STRESS RELATIONS IN SOCIALLY ISOLATED GROUPS. *J Pers Soc Psychol*. 1968;9(4):369-376. doi:10.1037/h0026088
27. Zuckerman M, Persky H, Hopkins TR, Murtaugh T, Basu GK, Schilling M. Comparison of Stress Effects of Perceptual and Social Isolation. *Arch Gen Psychiatry*. 1966;14(4):356-365. doi:10.1001/archpsyc.1966.01730100020004
